# A causal study of the phenomenon of ultrasound neurostimulation applied to an *in vivo* invertebrate nervous model

**DOI:** 10.1038/s41598-019-50147-7

**Published:** 2019-09-24

**Authors:** Jérémy Vion-Bailly, W. Apoutou N’Djin, Ivan Mauricio Suarez Castellanos, Jean-Louis Mestas, Alexandre Carpentier, Jean-Yves Chapelon

**Affiliations:** 1 LabTAU, INSERM, Centre Léon Bérard, Université Lyon 1, Univ Lyon, F-69003, Lyon, France; 2 Assistance Publique Hôpitaux de Paris, Hôpital Pitié-Salpêtrière, Neurosurgery department, Paris, F-75013 France; 3 Sorbonne Université, Paris, F-75005 France

**Keywords:** Neural circuits, Biomedical engineering, Acoustics

## Abstract

Focused ultrasound are considered to be a promising tool for the treatment of neurological conditions, overcoming the limitations of current neurostimulation techniques in terms of spatial resolution and invasiveness. Much evidence to support the feasibility of ultrasound activation of neurons at the systemic level has already been provided, but to this day, the biophysical mechanisms underlying ultrasound neurostimulation are still widely unknown. In order to be able to establish a clear and robust causality between acoustic parameters of the excitation and neurobiological characteristics of the response, it is necessary to work at the cellular level, or alternatively on very simple animal models. The study reported here responds to three objectives. Firstly, to propose a simple nervous model for the study of the ultrasound neurostimulation phenomenon, associated with a clear and simple experimental protocol. Secondly, to compare the characteristics of this model’s nervous response to ultrasound neurostimulation with its nervous response to mechanical and electrical stimulation. Thirdly, to study the role played by certain acoustic parameters in the success rate of the phenomenon of ultrasound stimulation. The feasibility of generating action potentials (APs) in the giant axons of an earthworm’s ventral nerve cord, using pulsed ultrasound stimuli (*f* = 1.1 MHz, *N*_*cycles*_ = 175–1150, *PRF* = 25–125 Hz, *N*_*pulses*_ = 20, *P*_*A*_ = 2.5–7.3 MPa), was demonstrated. The time of generation (TOG) of APs associated with ultrasound stimulation was found to be significantly shorter and more stable than the TOG associated with mechanical stimulation (*p* < 0.001). By applying a causal approach to interpret the results of this study, it was concluded that, in this model, the nervous response to focused ultrasound is initiated along the afferent neurons, in between the mechanosensors and the synaptic connections with the giant axons. Additionally, early results are provided, highlighting a trend for the success rate of ultrasound neurostimulation and number of APs triggered per response to increase with increasing pulse repetition frequency (*p* < 0.05 and *p* < 0.001, respectively), increasing pulse duration and increasing pulse amplitude.

## Introduction

Currently, the gold standard for the treatment of neurological conditions, when they are related to the central nervous system, is electrical deep brain stimulation (DBS) or direct cortical stimulation. These techniques have a high spatial resolution, which comes with a high degree of invasiveness, since they entail the insertion of electrodes in or on top of the cerebral cortex. More recently, mini-invasive or non-invasive techniques have emerged, such as transcranial magnetic stimulation (TMS) and transcranial direct current stimulation. In a practical guide published in 2012^1^ Fitzgerald and Daskalasis showed how high frequency repetitive TMS applied to the left dorsolateral prefrontal cortex was an effective treatment for patients suffering from major depressive disorders, and how this treatment could benefit from a method enabling brain structures to be targeted in a more accurate and reliable way^2^. If these promising results are confirmed, these techniques have the advantage of being much less invasive than DBS. However, they provide poor spatial resolution, and allow only superficial regions of the brain to be targeted, which presents major limitations in terms of treatment specificity. In this context, ultrasound could offer an alternative modality of neurostimulation overcoming both of the above limitations of current techniques. Indeed, ultrasound enable deep tissues to be targeted, with sub-millimetric spatial resolution. In addition, multi-element phased-array ultrasound transducers enable dynamic ultrasound beam steering using electronic focusing to target multiple tissue regions without requiring mechanical movement of the device.

In therapy, high intensity focused ultrasound (HIFU) have been mainly developed to destroy tissues using mechanical and/or thermal means and are proposed for the treatment of localized diseases (e.g. localized tumors^3–7^, kidney stones^8^). MR-guided focused ultrasound has also been proposed as a non-invasive method of thalamotomy for the treatment of Essential Tremor^9–11^. Low-energy ultrasound (LEUS) can be applied for drug-delivery enhancement in tumors^12,13^, BBB disruption^14^ and modulation of some cell dynamics^15,16^.

Between the 1950’s and 1970’s, much evidence of the feasibility of ultrasound activation of neurons at the systemic level was provided, notably by Fry *et al*.^17^ and Gavrilov *et al*.^18,19^. However, for decades, the medical ultrasound community seemed to lose interest in the study of ultrasound neurostimulation, instead focusing research efforts on the afore-mentioned successful applications. It is only recently that this field of study has seen renewed interest with several promising demonstrations of the feasibility of ultrasound-mediated induction of neuronal activity at the systemic level, using multiple animal models^20–23^.

While all these findings are very promising for future therapeutic applications, the biophysical mechanisms underlying the phenomenon of ultrasound neurostimulation are still widely unknown.

The interaction between ultrasound and neurons is difficult to explain using the Hodgkin-Huxley (HH) model alone^24^, which seems to reach its limits when studying this complex phenomenon. As highlighted in the literature^25^, a number of thermodynamic findings regarding the propagation of action potentials (APs) are not contained in the HH model, such as reversible heat exchange and modification of membrane length and thickness. Consequently, several research teams have postulated new models for the propagation of APs along an axon, aiming to complete or totally replace the classical HH model. As early as 1975, relying on the recently introduced concept of flexoelectricity, it was suggested that the AP could be modeled as a flexoelectric wave^26^. In this hypothesis, “it is admitted that the spherical deformation of planar membrane produces a flexoelectric polarization which results in depolarizing electric field”. Since the reverse effect is also true, similarly to piezoelectricity, this explains the propagation of a wave along the axon. In 2005, it was postulated that this propagating wave was actually a density wave (soliton)^27^. This hypothesis is backed up by the thermodynamics of phase transition within a lipid bilayer, which bring the necessary conditions (non-linearity and dispersion) for the propagation of a soliton in a medium. In 2010, a “continuum mechanics hypothesis” was proposed, in which ultrasound neuromodulation is the result of ultrasonic effects on the cerebro-spinal fluid, which is Newtonian, and the viscoelastic membrane of neurons, which is non-Newtonian, eventually leading to alteration of membrane conductance^28^. More recently, another model was developed wherein the key to ultrasound neurobiological effects lays within the alteration of membrane conductance: the neuronal intramembrane cavitation excitation (NICE) model^29,30^. In the NICE model, bubbles oscillating within the lipid bilayer act as sonophores modulating the membrane capacitance, which induces intramembrane electric currents and eventually leads to the triggering of an AP.

Considering the various demonstrations of the feasibility of ultrasound neurostimulation/neuromodulation at the systemic level mentioned above, and the different models postulated to explain the phenomenon, it is also necessary to work at the cellular level, or on very simple animal models, in order to establish a clear and robust causality between acoustic parameters of the excitation and neurobiological characteristics of the response. Perfectly controlling the phenomenon on a simple nervous model can be the first step towards translating promising preclinical findings into actual clinical applications of ultrasound neurostimulation.

Hence, the first objective of this study was to propose a simple nervous model for the study of ultrasound neurostimulation/neuromodulation phenomena, associated with a clear and simple experimental protocol enabling further investigation into various combinations of acoustic parameters. The animal model should ideally be as stable as possible, involving linear nervous structures or really simple networks, preferably *in vivo*, as well as available and affordable worldwide. For these reasons, the authors chose to work on the ventral nerve cord of the common earthworm (*lumbricus terrestris*), which presents in particular three giant axons: the Medial Giant Fiber (MGF) and the two Lateral Giant Fibers (LGF)^31^.

Once the feasibility of the ultrasound stimulation of the nervous model was demonstrated, ultrasound neurostimulation (UStim) was compared with two other modalities of neurostimulation for which theoretical knowledge is available in the literature: mechanical stimulation (MStim) and electrical stimulation (EStim). Some characteristics of the nervous response to UStim were studied in the proposed earthworm model, and compared to those obtained with standard MStim and EStim. This comparative study aimed to build new knowledge on the biological basis of the nervous response to UStim, using a causal approach.

Finally, the effects of some LEUS parameters on the success rate of the UStim phenomenon were also investigated.

## Results

### Focused ultrasound can repeatedly trigger the generation of APs in earthworm giant axons

**Figure 1:**
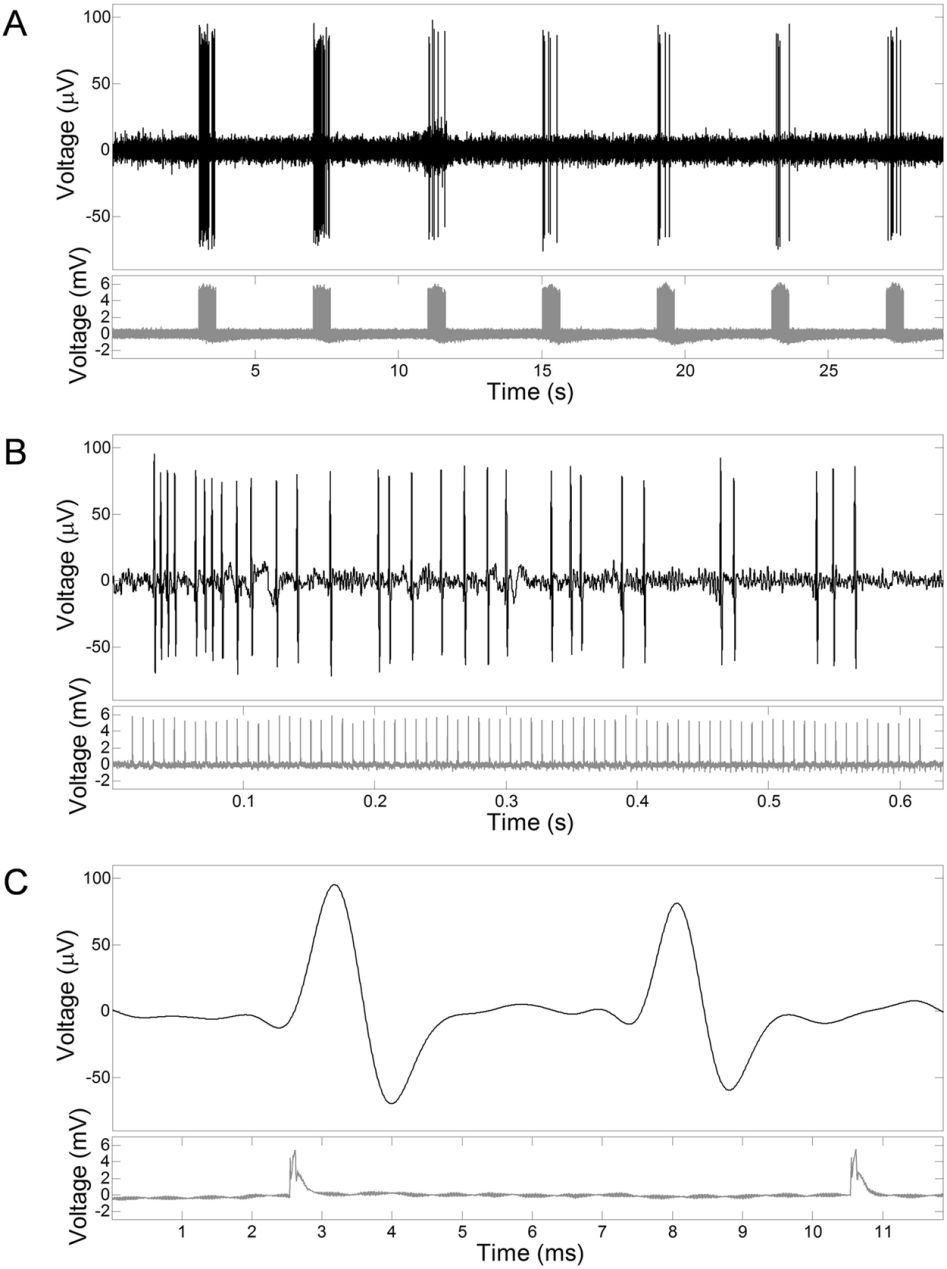
Example of the structure of a LEUS sequence and its associated nervous response. Different timescales are displayed, in a decreasing order: (**A**) the scale of a whole sequence of LEUS bursts, (**B**) the scale of a burst of LEUS pulses and (**C**) the scale of the period of repetition of LEUS pulses.

Of the animals tested (*n* = 84), 92% presented a responsive nervous system after anesthesia. Of those, nervous responses to LEUS exposures were successfully observed in 88% of cases. A typical pattern of response to a LEUS sequence is provided in Fig. 1. On the scale of a LEUS sequence, it was observed that once the nerve had responded to a first LEUS burst, the following bursts were likely to trigger nervous responses as well. On average, the number of APs per burst would decrease over time, until there was no more response, as illustrated by Fig. 1A. Waiting a significant amount of time (1–20 minutes) without sending LEUS bursts, or slightly displacing the focal area along the nerve (1–2 mm in longitudinal direction), would enable new responses to be triggered. Eventually, after a period of time ranging from 30 minutes to several hours, depending on the animal, the nerve would stop responding regardless of the localization of the focal spot or the period of rest.

On the time scale of a LEUS burst, it was observed that on average the time difference between two consecutive AP recordings increased over time, as illustrated by Fig. 1B.

### Electrical stimulation concurrently triggers MGF- and LGF-associated APs with a repeatable time of generation

**Figure 2:**
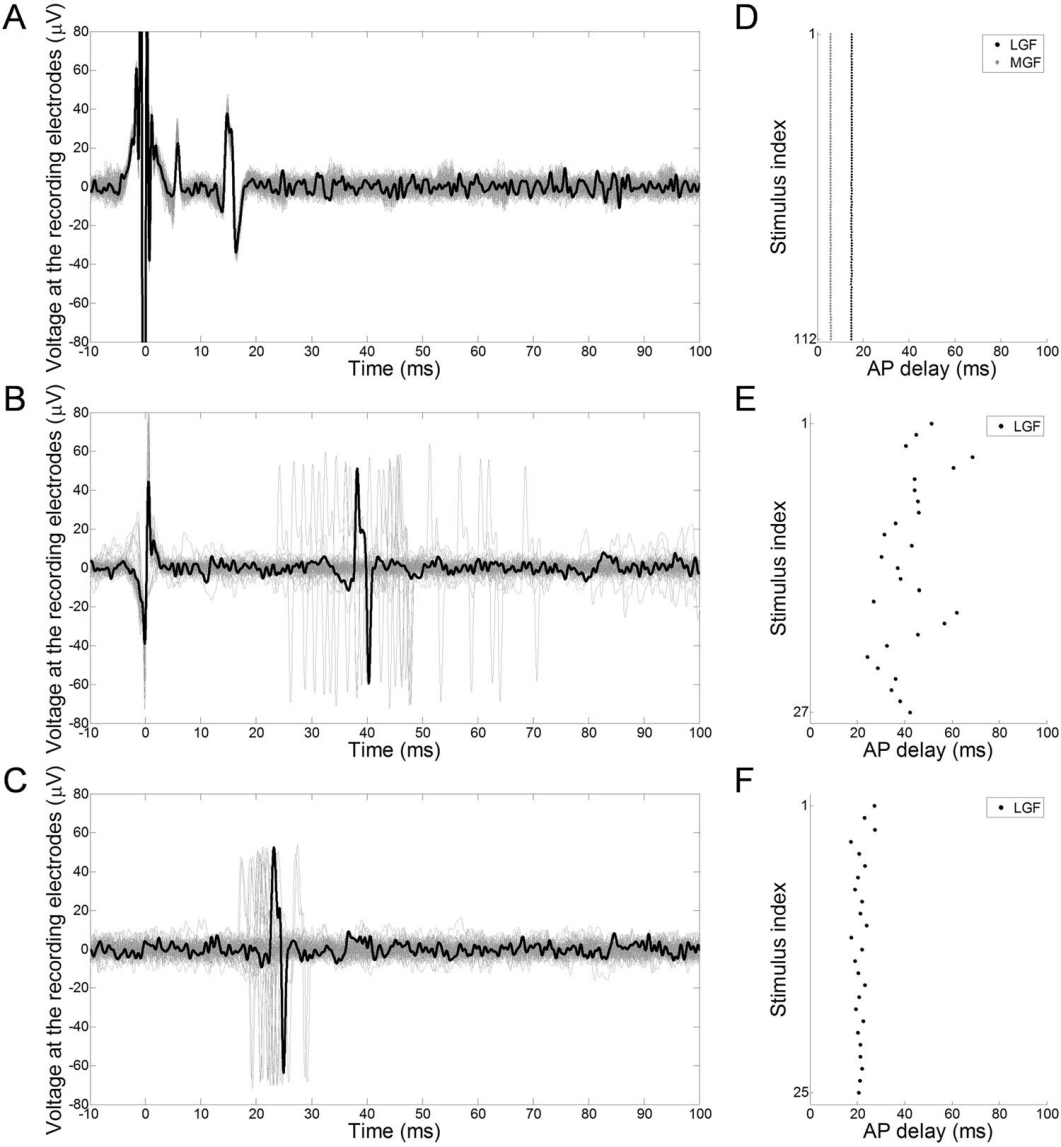
Superposition of the response signals recorded in the ventral nerve cord following repeated instances of (**A**) EStim, (**B**) MStim and (**C**) UStim. Time reference is the stimulus onset. A random waveform is highlighted in black in order to identify the general shape of an AP. (**D**–**F**) Present the successive arrival delays corresponding to the APs displayed in (**A**–**C**), respectively. Data for UStim and MStim were recorded during the same trial.

The nervous response signal to EStim, regardless of the region of stimulation on the animal, systematically comprised a single MGF-associated AP and a single LGF-associated AP (Fig. 2A). In a minority of cases, a slight change (1–3 mm, longitudinally) to the position of either stimulating or recording electrodes was necessary before being able to observe both types of APs. The respective delays between the EStim onset and the times of arrival of MGF-associated and LGF-associated APs were stable from one response to another. In the example presented in Fig. 1A, the conduction velocities (CVs) of APs along both types of axons were measured as 16.6 m/s [16.5–16.8] and 9.0 m/s [9.0–9.1] in MGF and LGF, respectively. From these values, the estimated TOPs over an axonal distance of 8.4 cm were 5.1 ms [5.0–5.1] and 9.3 ms [9.2–9.4] for MGF and LGF, respectively, while the delays of arrival of the AP were 6.4 ms [6.3–6.4] and 11.7 ms [11.7–11.7], leading to TOGs of 1.3 ms [1.3–1.3 ms] and 2.4 ms [2.3–2.4 ms] for MGF and LGF, respectively.

### Mechanical stimulation exclusively triggers one type of AP with a variable time of generation

The nervous response signal to a manual MStim at the surface of the earthworm’s skin was a variable number of either MGF-associated or LGF-associated APs, but never of both types of APs (Fig. 2B). A MStim administered in the anterior third of the animal would exclusively trigger the generation of MGF-associated APs, whereas a MStim administered in the posterior third of the animal would exclusively trigger the generation of LGF-associated APs. A MStim administered in the medial third of the animal occasionally triggered the generation of either type of AP, but did not lead to any nervous response in the majority of cases. In the example presented in Fig. 2B, the mean conduction velocity of APs along LGF was measured at 8.2 m/s. From this value, the mean estimated TOP over an axonal distance of 7.5 cm was 9.2 ms, while the arrival delay of the AP was 42.2 ms [35.3–45.7], leading to a TOG of 33.0 ms [26.1–36.6].

### LEUS stimulation exclusively triggers one type of AP with a variable time of generation

The nervous response signal to UStim was a variable number of either MGF-associated or LGF-associated APs, but never of both types of APs (Fig. 2C). A regional dependence could be highlighted, similar to that observed with MStim: an UStim administered in the anterior third of the animal would exclusively trigger the generation of MGF-associated APs, whereas an UStim administered in the posterior third of the animal would exclusively trigger the generation of LGF-associated APs. An UStim administered in the medial third would trigger no nervous response in the majority of cases. In the example presented in Fig. 2C, the conduction velocity of APs along LGF was measured at 8.1 m/s [8.0–8.3]. From this value, the estimated TOP over an axonal distance of 7.5 cm was 9.2 ms [9.1–9.3], while the arrival delay of the AP was 21.2 ms [20.3–22.5], leading to a TOG of 11.8 ms [11.2–13.1].

### UStim-associated time of generation is shorter and more stable than MStim-associated time of generation

**Figure 3:**
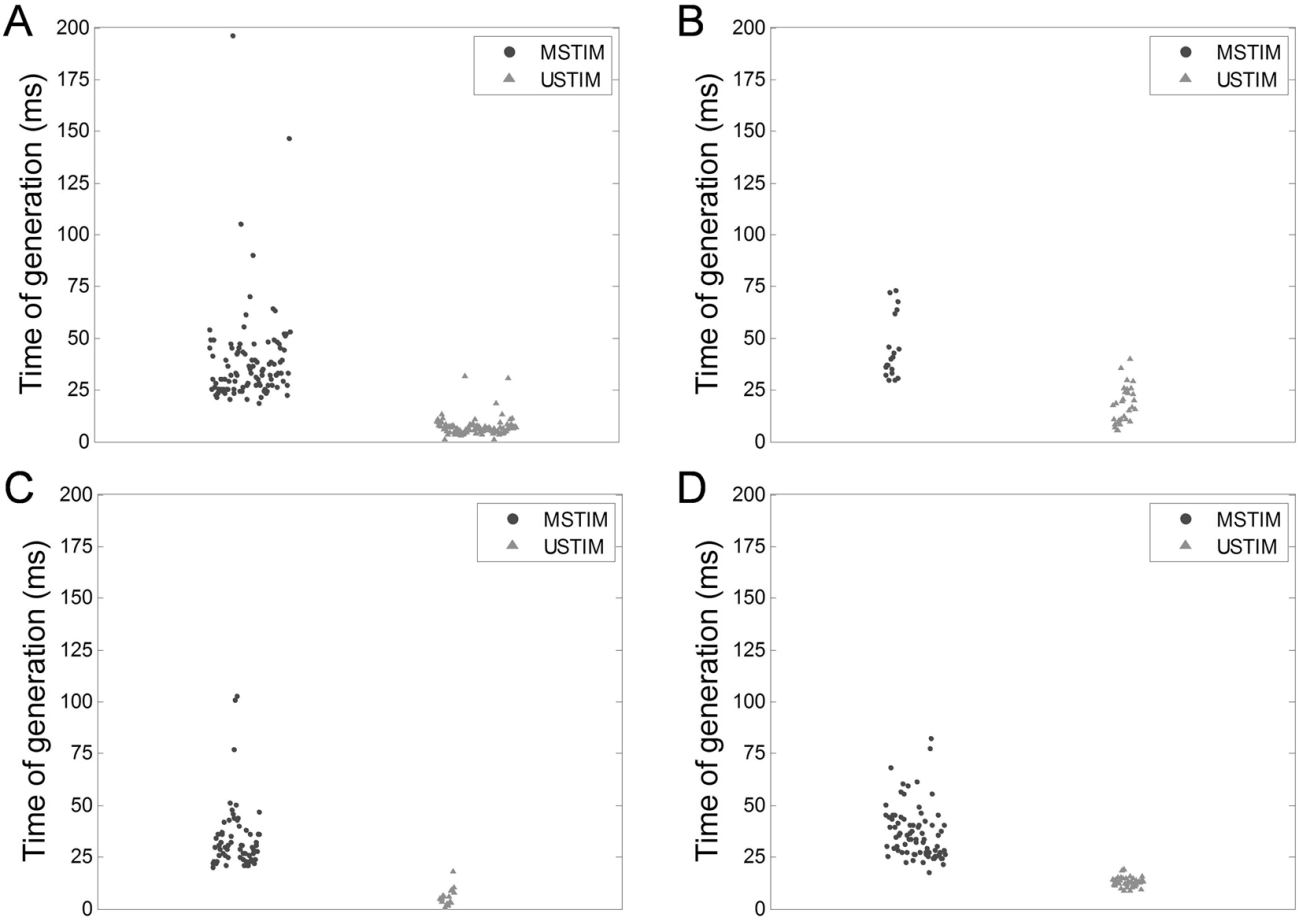
Comparative trial between MStim and UStim. Distributions of TOG associated with MStim (black dots) and UStim (grey triangles), over 4 trials (**A**–**D**). In every trial, the animal received alternatively MStim and UStim, administered in the same location. On the charts, the horizontal component of the distribution is only meant to faciliate the points visualization for the reader, hence the horizontal axis is not associated with any unit or meaning.

Further investigations directly compared the characteristics of these nervous responses by alternatively administering MStim and UStim in the same animal location. The respective distributions of TOGs associated with both modalities of stimulation, over every trial, are presented in Fig. 3. These investigations revealed that, in every trial, the TOG of APs associated with UStim were significantly shorter and more stable than the TOG of APs associated with MStim (***p < 0.001). Statistical data for each individual trial are presented in Table 1.

**Table 1:** Statistical data for the comparative trials between MStim and UStim.

Trial	TOG associated with MStim				TOG associated with Ustim				p-value*
	Median value	Q1	Q3	Number of measures	Median value	Q1	Q3	Number of measures	
1	32.5	26.0	45.0	107	6.4	4.9	7.8	105	***
2	30.1	25.1	36.6	19	17.2	10.6	23.9	30	***
3	40.6	33.6	58.1	63	4.7	2.5	7.0	20	***
4	35.1	27.6	43.6	80	12.9	12.0	14.4	41	***

*H0: the distribution of the TOG is the same for the MStim and UStim groups.

### The success rate of LEUS neurostimulation increases with the PRF of the pulsed ultrasound sequences

**Figure 4:**
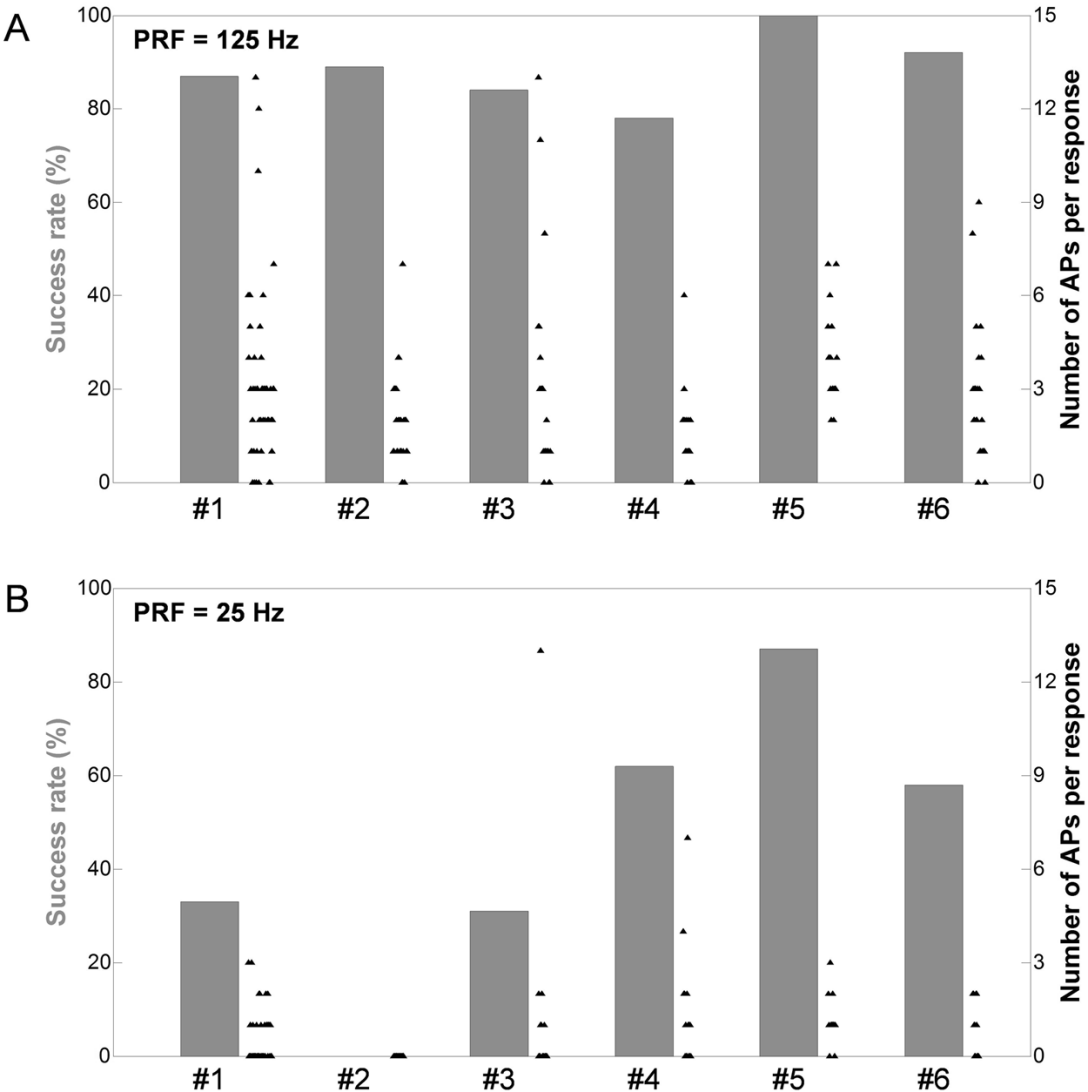
Influence of PRF over success rate of ultrasound neurostimulation. Success rates (grey bars) and number of APs triggered per response (each black triangle represents a stimulus, including those which did not trigger any nervous response), associated with two types of pulsed ultrasound sequences, with respective PRF of (**A**) 125 Hz and (**B**) 25 Hz. With the exception of their PRF, the two types of ultrasound sequences shared the same acoustic parameters (*f* = 1.1 MHz, N_cycles_ = 175, *P*_*A*_ = 6.6 MPa, *N*_*pulses*_ = 20). For every trial (*n* = 6), the number of instances of each stimulus and their order of administration was randomized.

The effect of the pulse repetition frequency (PRF) on the success rate was investigated by alternatively administering, to a given animal, two types of pulsed LEUS stimuli, with respective PRFs of 25 and 125 Hz. Figure 4 presents the respective success rates associated with both types of sequences. It was observed that over every trial, the success rate associated with the high PRF was significantly higher than the success rate associated with the low PRF (*p < 0.05). Furthermore, in every trial except one (trial No. 4, *p* > 0.1), the number of APs triggered per response tended to be significantly higher for the high PRF stimuli (***p < 0.001).

### The success rate of ultrasound neurostimulation increases with the amplitude and duration of the ultrasound pulses

**Figure 5:**
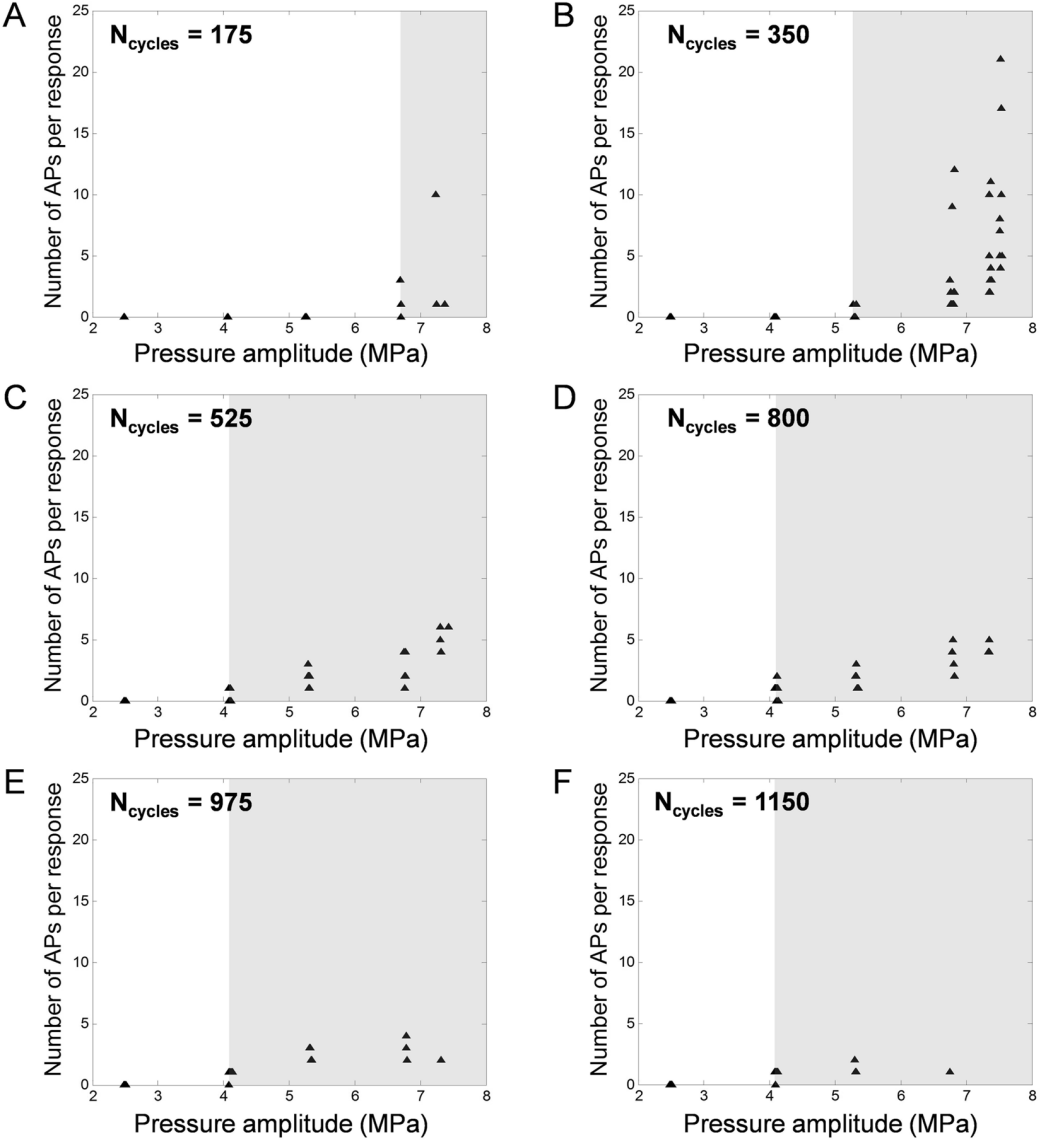
Numbers of APs triggered per response for a range of pressure amplitudes (2.5; 4.0; 5.2; 6.6; 7.1; 7.3 MPa). Each black triangle represents a stimulus, including those which did not trigger any nervous response. Each chart (**A**–**F**) corresponds to a different number of cycles per pulse (175; 350; 525; 800; 975; 1150). The white-grey boundaries indicate the threshold under which no nervous response could be triggered. All sequences shared the same harmonic frequency (*f* = 1.1 MHz) and number of pulses (*N*_*pulses*_ = 20).

The effect of varying the ultrasound pulse durations and amplitudes on the neural responses was investigated and enabled the identification of the minimum thresholds above which UStim starts to induce nervous responses (Fig. 5). No neural response could be observed below a pressure amplitude *P*_*A*_ = 4.0 MPa in the range of pulse durations between 0.16 and 1.05 ms (*N*_*cycles*_ = 175–1150). In addition, the threshold amplitude over which a pulsed ultrasound sequence starts to induce nervous responses tends to decrease with increasing pulse duration.

## Discussion

The observations made from the nervous responses associated with EStim and MStim are consistent with what was expected, on the basis of the literature and preliminary studies.

### Dynamics of the nervous response to EStim

**Figure 6:**
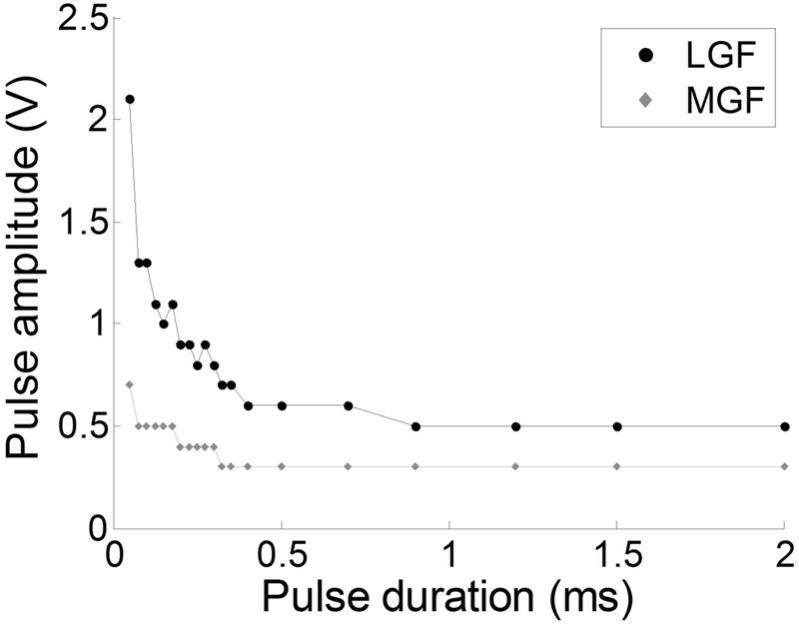
Strength-duration curve built over an EStim trial. Each point corresponds to the amplitude threshold above which, for a given electric pulse duration, an electrically evoked AP can be triggered. For the same pulse duration, the LGF-associated threshold (black dots) is intrinsically higher than the MGF-associated threshold (grey diamonds). Above a certain pulse duration, the activation threshold reaches a plateau.

It is established that an electrical stimulus can depolarize the membrane of an axon, leading to the generation of one to several APs. In previous works, the authors dedicated a large amount of time studying the responses of the earthworm nervous system to an electrical pulse administered through invasive electrodes. For a given pulse duration, there is a pulse amplitude threshold above which a nervous response of the giant axon will be triggered. According to the literature on the subject and experimental verification, the threshold associated with LGF is intrinsically higher than the threshold associated with MGF (Fig. 6). Hence, the fact that EStim invariably triggered both MGF- and LGF-associated APs can be explained by the use of a pulse amplitude (8 V) much higher to the experimentally assessed range of LGF-associated threshold (~2 V). In parallel, the very short pulse duration (50 µs), relative to the refractory period of the ion channels (several milliseconds), can explain the triggering of only one instance of each type of AP.

The depolarization of the membranes of the giant axons can be considered as instantaneous, with regard to the timescale of the AP propagation on distances such as used in the presented trials (centimeter scale). Besides, the stability of the conduction velocities of giant axons over time was experimentally assessed, and it was determined that they could be considered to be steady over the duration of a trial. This hypothesis was confirmed during the trials with EStim, since the delays between the stimulus onset and the times of arrival of the APs were steady (Fig. 2D).

### Dynamics of the nervous response to MStim

The locus of interaction between a mechanical stimulus at the surface of the earthworm skin and its internal nervous system (which can be viewed as a ‘point of entry’ of the information) is the mechanosensitive end of the afferent neurons. The activation of a mechanosensor leads to the generation of APs along the afferent nerve, or afferent spiking (Phase I)^32^. This afferent spiking propagates inward, to the synaptic connection with the giant axons (Phase II). The synaptic connection then translates this spiking into postsynaptic potential activity and eventually to the generation of an AP in the giant axon (Phase III). The incompressible durations of these different phases^33^ explain why non-negligible TOGs have been observed by recording APs in the giant axons during MStim trials. Furthermore, these different phases of translation/propagation of the sensory information may all present a variability of their own, which is consistent with the TOGs measured during MStim being variable from one neural response to another. The time latency associated with the whole process was specifically studied by Moore, and was found to be equal to 6 ms. However, the experimental conditions of this past study (animal preparation, stimulation protocol) greatly differed from those of the present study, hence the respective results must be compared with caution.

The spatial polarity of the MStim-associated excitability map (MGF only activated by anterior mechanical stimulation and LGF only activated by posterior mechanical stimulation) is consistent with the literature on the subject^31,33^. Giant axons are the key supporting structures in the rapid escape response of the earthworm^32^. If the animal encounters a threat coming from the front, it triggers a rapid withdrawal backward, mediated by MGF. Symmetrically, a threat coming from behind induces the activation of LGF, which sends APs propagating upstream and triggering the appropriate muscle contraction to move forward.

The structures responsible for the polarity in the excitability map of the earthworm body are the synaptic connections between afferent neurons and giant axons. There are a fixed number of them per segment but the strength of their synaptic connection is modulated along the ventral nerve cord^32^. This last point is of significant importance in the next section of the discussion.

### Deductions on the locus of interaction between ultrasound and the earthworm nervous system

Firstly, the fact that the excitability map associated with UStim presents the same polarity as that associated with MStim suggests that the locus of interaction between ultrasound and the earthworm nervous system is situated upstream the synaptic connection between afferent neuron and giant axon. In other words, focused LEUS do not directly activate the giant axons. Otherwise, given the various parameters tested in the presented study, it is reasonable to believe that both types of APs (MGF- and LGF-related) would have been triggered, similarly to responses induced by EStim.

Secondly, the fact that the TOGs were significantly shorter with UStim than with MStim indicates that the nervous response associated with UStim is initiated somewhere downstream the point of initiation of the nervous response associated with MStim, which is the mechanosensitive end of the afferent neuron. This interpretation is also reinforced by the TOGs being more stable from one response to another with UStim. Indeed, the shorter the nervous pathway between the locus of stimulation and locus of recording, the fewer sources of variability in the timing of the neural responses. In other words, focused LEUS exposures do not simply activate the mechanosensors as a mechanical stimulus would do, but rather interact at a deeper level.

By combining the two previous conclusions, a close estimation of the locus of interaction of ultrasound with the earthworm nervous system can be obtained, somewhere along the afferent neurons, excluding the mechanosensor and including the synaptic connection.

### Deductions from parametric studies on the success rate of ultrasound neurostimulation

The trends highlighted in the afore-presented parametric studies regarding the influence of *PRF*, pulse amplitude and duration on the success rate of the LEUS neurostimulation phenomenon are encouraging. Further investigations are however necessary to repeat these trials and assess the influence of other acoustic parameters before being able to draw any conclusions on the biophysical mechanisms involved. In particular, the trials relating to the identification of amplitude-duration thresholds must be repeated, using finer pulse amplitude steps, to confirm the existence of a plateau, which recalls some aspects of a strength-duration curve.

## Conclusion

The feasibility of using pulsed focused ultrasound to induce nerve response from the anesthetized earthworm was demonstrated. Ultrasound stimuli were delivered at both animal extremities, or ventrally in the median third of its length. The form of nerve response evoked was serials of action potentials in the giant fibers. The response of our study model to alternative modalities of stimulation was studied. We applied a causal approach to interpret on one hand the respective sensory fields associated with these different modalities, and on the other hand the delays between stimulus onset and detection of an action potential in the giant fibers. Two strong interpretations could be built from these observations. Firstly, the burst of focused ultrasound used in our study do not interact directly with the giant fibers but rather activate the afferent pathway at a level high enough to eventually trigger a spiking in the giant fibers. Secondly, the bursts of focused ultrasound in our study do not interact with the mechanosensitive end of the segmental nerves such as a tactile stimulus would do, but rather interact somewhere downstream on the afferent pathway. In conclusion, our hypothesis is that during the phenomenon of ultrasound neurostimulation highlighted in our studies, the structures functionally responding to the ultrasound stimulus are the segmental afferent nerves. This study and its conclusions illustrated the relevance of the present nervous model to study the phenomenon of ultrasound neurostimulation.

## Methods

### Animal preparation

All animal experiments undertaken for the present study were conducted in strict accordance with the legal conditions of the French National Ethics Committee of Reflection on Animal Experimentation (CNREEA). Common earthworms (*lumbricus terrestris*) were purchased in a local fishing shop and kept at 5 °C in containers filled with enriched soil. They were utilized within the two following weeks, otherwise they were released back to the wild. Earthworms were anesthetized prior to each trial by being immerged in a 10% ethanol solution^34^. Times of immersion ranged from 8 to 15 minutes, depending on the size of the animal. The criterion chosen to end the immersion phase was the absence of response to mechanical stimuli at both ends of the animal. Earthworms were carefully rinsed and dried before being set on the neurostimulation platform. Throughout the trial, whenever the animals exhibited muscular artifacts biasing the measures or showing signs of awaking, they were put back in the anesthetic solution for 1 to 5 minutes, until the above-mentioned criterion of complete anesthesia was met. Nerve functionality was asserted through either mechanical or electrical stimulation. Whenever both modalities of stimulation failed to trigger a nervous response, the animal was not included in the study.

### Neurostimulation platform

**Figure 7:**
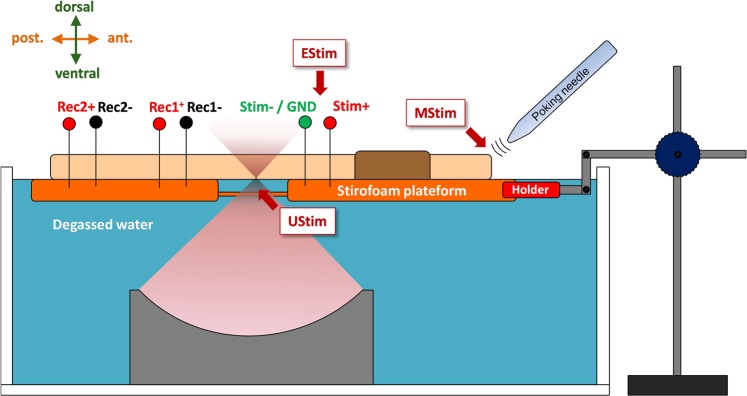
Schematic representation of the experimental set-up, gathering the following functionalities: (i) Recording of action potentials in the giant axons; (ii) Mechanical stimulation; (iii) Electrical stimulation and (iv) LEUS stimulation. For more clarity, each modality of stimulation is represented as if applied to a distinct region of the animal, although during comparative trials all the stimuli were administered at the same location.

A neurostimulation platform was developed in-house which included a tank of degassed water with an immersed LEUS transducer ballasted at the bottom for upward ultrasound exposures (Fig. 7). The earthworm lay on a homemade electrically isolated watertight bed (polystyrene covered with insulated tape) allowing the insertion of multiple electrodes on its upper side while its lower side was placed in contact with the surface of the degassed water. The bed had a hole at its center (2 cm in diameter) to provide acoustic coupling between the LEUS transducer and a portion of the earthworm body. A mechanical arm with 3 degrees of freedom in translation allowed moving precisely the bed in order to adjust the focal region relatively to the animal. A multichannel electrophysiology system was used (PowerLab 8/35, ADIntruments, France). This system allows recording axons electrical signals on up to 8 analog input channels in parallel with a maximum amplitude resolution of 16 Bits (63 nV within a ±2 mV dynamic range) and a maximum sampling rate of 40 kHz (200 kHz when using 1 or 2 input channels only). The system also allows generating electric stimulations with 2 analog output channels (programmable waveforms, amplitude range: ±10 V). The softwares used for the signals data display and post-analyses were LabChart Pro v8 (ADIntruments, France) and MATLAB (R2014a, The Mathworks, Inc., USA). The described platform gathered the following functionalities which are further described below: i) Recording of action potentials in the giant axons; ii) Mechanical stimulation; iii) Electrical stimulation and iv) LEUS stimulation.

### Recording of action potentials in the giant axons

Because of their exceptionally large diameter, MGF and LGF produce action potentials with high depolarization voltage amplitude. At this level, it is possible to detect the passing of an action potential by simply inserting macroscopic electrodes through the animal, with no necessity for them to be in the close vicinity of the ventral nerve cord. In the presented study, the best compromise between the data acquisition quality and the procedure safety was found by inserting the electrodes at one quarter of the width of the animal in order to avoid any risk of hemorrhage, which could biased the stability of the nervous model during the trial. All data presented in this document are filtered through a bandpass filter (120–1200 Hz). These cutoff frequencies were tuned to provide a steady baseline and to optimize the SNR.

Two pairs of electrodes were inserted into the animal. The 4 electrodes were aligned in longitudinal direction and spaced 1 cm apart from each other. The 2 anodes were spaced 2 cm and placed closer to the region of stimulation than their associated cathode (Fig. 7).

At rest, the voltage recorded by a pair of electrodes was null in average. If an action potential was triggered at the region of stimulation, the voltage would display a short biphasic event, such as the example provided in Fig. 1C (upper chart). The positive and negative peaks respectively correspond to the passage of the AP at the level of the anode and cathode. Using two sets of electrodes allowed to precisely compute the conduction velocity characteristic of both type of giant axon. The electrodes recorded APs propagating through both MGF and LGF, and unless they were overlaid, were easily distinguishable on the basis of their amplitude, shape and propagation speed.

### Mechanical stimulation

Mechanical stimulation was performed by manually poking the animal with a needle at the surface of its skin. A dedicated channel on the recording device measured the voltage between the needle and a reference electrode coupled with the animal in order to create an artifact when the needle was in contact with the animal’s skin. The positive peak of this artifact was used as a time reference for the mechanical stimulus onset.

### Electrical stimulation

Electrical stimulation was performed by inserting 2 electrodes into the animal and sending a short electrical pulse (50 µs, 8 V). The electrical pulse naturally created an electromagnetic artifact on the signal measured by the recording electrodes, which was used as a time reference for the electrical stimulus onset.

### Ultrasound stimulation

A mono-element spherically focused piezoelectric transducer (PZ28, Ferroperm, Kvistgaard, Denmark) presenting a central frequency of 1.1 MHz and a radius of curvature of 50 mm was used. The driving signal was built from two function generators (AFG1062 and AFG3102, Tektronix, France) and amplified by a 50 dB amplifier. The main ultrasound sequences used in this study consisted of a series of pulsed ultrasound bursts (*f* = 1.1 MHz, *N*_*cycles*_ = 175, *PRF* = 125 Hz, *N*_*pulses*_ = 20). The waveform of the electric driving signal was controlled with an oscilloscope (Picoscope 3000 Series, Pico Technology Ltd., UK). Acoustic pressure at the focal spot was calibrated with an optical hydrophone (FOPH 2000, RP Acoustics e.K., Germany). The levels of pressure amplitudes applied with LEUS exposures ranged from 2.5 to 7.3 MPa in degassed water. The ultrasound device was placed in the bottom of a tank filled with degassed water in such a way that the focal point of the transducers was included in the surface plan of the water (Fig. 7). The earthworm was laid out and pinned with the electrodes on a Styrofoam platform held above the water, on its ventral side, a medial portion of its body, or alternatively one of its ends, being immerged 1 to 3 mm under the surface of the water.

### Definition and calculation of the AP-associated arrival delay, TOP and TOG

The arrival delay Δt associated with an evoked AP was defined as the time difference between the stimulus onset and the peak of the AP. The time of propagation (TOP) associated with an AP was defined as the time necessary for the AP to propagate along the axonal distance between the point of stimulation and the recording anode. The TOP was calculated from the measurement of this distance and the mean value of the conduction velocity during the considered trial. Instantaneous values of conduction velocity were deduced from the time difference between the detection of the AP at two distinct recording sites. The time of generation (TOG) associated with an AP was defined as the difference between the arrival delay and the time of propagation: TOG = Δt − TOP.

### Definition of the success rate

The success rate of a given type of stimulus was defined as the number of instances having led to a nervous response divided by the total number of instances administered over the trial.

### Comparative study of MStim and UStim dynamics

To compare the MStim-associated TOGs with the UStim-associated TOGs, trials (*n* = 4) were performed where a given animal alternatively received mechanical and ultrasound stimuli, administered in the same location. For every response to a stimulus, the TOG was calculated and included in the group of data corresponding to its associated modality of stimulation.

### Parametric study of the PRF

To study the effect of the PRF of the LEUS sequence on the success rate, trials (*n* = 6) were performed wherein a given animal was exposed to two types of pulsed ultrasound sequences respectively presenting a high PRF of 125 Hz and a lower PRF of 25 Hz, all other UStim parameters being identical (*f* = 1.1 MHz, *N*_*cycles*_ = 175, *P*_*A*_ = 6.6 MPa, *N*_*pulses*_ = 20). UStim sequences were alternated in a random order. The value of the high PRF was chosen because it corresponds to a pulse repetition period of 8 ms, which is twice as high as the mean value of the refractory period of the giant axons. In this way, it was possible to avoid bias in the analysis of the number of APs triggered by a pulsed ultrasound sequence, by providing enough time for the membrane ion channels to go back to their resting state between two pulses.

### Parametric study of the amplitude and duration of pulses

To study the effect of the characteristics of LEUS pulses on the success rate, one trial was performed to identify the thresholds, in terms of pulse amplitude and pulse duration, above which a pulsed ultrasound stimulus starts to induce nervous responses. Six duration values (*N*_*cycles*_ = 175; 350; 525; 800; 975; 1150) and six amplitude values (*P*_*A*_ = 2.5; 4.0; 5.2; 6.6; 7.1; 7.3 MPa) were investigated. For every pulse duration (in increasing order) decreasing pulse amplitudes were tested until one was identified as having never induced any nervous response over more than 5 attempts.

### Assessment of results

To assess the TOG associated with each modality of stimulation, groups of APs generated within a relatively short period of stimulation (~2 mn) were considered. The assumption of normality and standard deviation was inspected for the TOGs by performing a Jarque-Bera test and could not be justified in all of the groups. Hence, quantitative results were presented as Median Value [1st quartile–3rd quartile]. Statistical significance for the comparison between MStim and UStim groups of TOG values was assessed using a Wilcoxon–Mann–Whitney test (H0: the distribution of the TOGs is the same for the MStim and UStim groups). Statistical significance for the comparison, within a given trial, between the number of APs triggered by high-PRF stimuli and low-PRF stimuli was assessed using a Wilcoxon–Mann–Whitney test. Statistical significance for the comparison between high-PRF- and low-PRF groups of success rate values was assessed using a Wilcoxon–Mann–Whitney test for paired samples (H0: the mean difference between the two measured values of success rate is null). The statistical analyses were performed using MATLAB (R2014a, The Mathworks, Inc., USA) and the BiostaTGV platform (https://biostatgv.sentiweb.fr/, UMR S 1136, INSERM, UPMC, France).

## Data Availability

The datasets generated and/or analyzed during the present study are available from the corresponding author subject to reasonable request.
